# Impact of Clubhouses in Reducing Psychiatric Readmission Risk

**DOI:** 10.7759/cureus.80559

**Published:** 2025-03-14

**Authors:** Shama Faheem, Marianne Lyons, Melissa Moody, Stacey Sharp, James White

**Affiliations:** 1 Psychiatry, Detroit Wayne Integrated Health Network, Detroit, USA; 2 Psychology, Detroit Wayne Integrated Health Network, Detroit, USA; 3 Behavioral Health, Detroit Wayne Integrated Health Network, Detroit, USA

**Keywords:** clubhouse, psychiatric hospitalization, psychosocial, readmission, recidivism, rehabilitation

## Abstract

Introduction

Individuals with severe mental illness (SMI) have complex needs that can contribute to repeated psychiatric admissions if not addressed adequately. Interventions that can reduce psychiatric recidivism are an area of interest and focus given the high readmission rates for this population. A clubhouse is a community-based service dedicated to supporting and empowering people living with mental illness. While there have been studies on the rehabilitative and recovery aspects of clubhouse participation, limited research has focused on its direct impact on clinical outcomes, particularly in reducing psychiatric readmission rates.

Objective

The period following discharge from a psychiatric hospital is considered a "risk period" for readmission. For the purpose of this study, the risk period is defined as 90 days following a discharge from a psychiatric hospital. As part of this retrospective study, we identified "at-risk" individuals with a recent psychiatric admission, who started clubhouse during that period. We hypothesized that clubhouse participation during the high-risk post-discharge period would be associated with a statistically significant reduction in 90-day psychiatric readmission rates compared to pre-enrollment hospitalization rates. The objective of the study was to identify if clubhouse participation resulted in a reduction in hospital readmission as compared to the general 90-day readmission risk in SMI individuals during the risk period.

Methodology

The Detroit Wayne Integrated Health Network (DWIHN), a Prepaid Inpatient Health Plan (PIHP), provided claims data for clubhouse participation and inpatient psychiatric admissions. Clubhouse members were narrowed to include a subgroup that had at least one psychiatric admission in 90 days before starting clubhouse. Their psychiatric readmission rates were assessed for the 90 days after starting clubhouse. Statistical significance was established at 0.05, and a two-tailed t-test was performed to determine if the reduction in psychiatric admissions was related to clubhouse participation. Ninety-day readmission rates were calculated for clubhouse members and compared to general DWIHN 90-day readmission rates.

Results

An eightfold reduction in rehospitalization was noted in the subsequent 90 days for members who started and engaged in clubhouse during the risk period. When compared to DWIHN's general population, this was approximately a 50% lower rate of 90-day hospital readmission rate.

Conclusion

Clubhouses have an effective role not only in rehabilitation but also in reducing psychiatric readmissions during the high-risk period.

## Introduction

Individuals with severe mental illness (SMI) have complex conditions and multiple unmet needs that often result in low outpatient follow-up and a lack of appropriate treatment [1]. Many of these unresolved needs that have not been addressed through outpatient treatment often lead to psychiatric admissions and, in some cases, frequent readmissions, contributing to recidivism [2]. A study categorizing unmet needs and previous psychiatric admissions as main risk factors for recidivism identified the overall rates of psychiatric readmissions for their population within 30 days, 90 days, and one year after discharge to be 21.21%, 40.40%, and 61.61%, respectively [2]. Interventions focused on reducing psychiatric readmission rates have highlighted the importance of discharge planning and support involvement as well as easier access to mental health services including within residential settings, improving medication adherence after discharge, and an overall improvement in the crisis services [2,3]. To address outpatient treatment refusal in this high-need and high-risk group, outpatient court-ordered treatment is sometimes considered to improve treatment compliance. Despite being a restrictive intervention, it has been shown to work in specific populations on reducing hospital readmissions and total hospital days given that the court orders are sustained and combined with intensive treatment and are not used as a replacement [4]. Ideally, more therapeutic, engaging, and less restrictive interventions, such as clubhouse, are preferred if able to achieve the desired outcome of decreasing psychiatric readmissions in the high-risk group.

Clubhouses are models of psychosocial rehabilitation that offer a "collaborative, restorative environment" where members get a chance to connect with each other and recuperate through opportunities from employment, socialization, education, skill development, housing, and improved wellness [5]. There have been multiple studies done on the rehabilitative aspects of clubhouses in promoting employment, advocacy, social integration, and a positive sense of well-being [6-8] which are not only observed by the members themselves but are appreciated by the families as well [9]. Though there are studies that have shown a reduction in hospitalizations and psychiatric symptoms for members attending clubhouses [10-14], these studies are either old or usually limited in number and quality.

Objective

The period following discharge from a psychiatric hospital is considered a "risk period" for readmission. For the purpose of this study, the risk period is defined as 90 days following a discharge from a psychiatric hospital. As part of this retrospective study, we identified "at-risk" individuals with a recent psychiatric admission, who started clubhouse during that period. We hypothesized that clubhouse participation during the high-risk post-discharge period would be associated with a statistically significant reduction in 90-day psychiatric readmission rates compared to pre-enrollment hospitalization rates. The objective of the study was to identify if clubhouse participation resulted in a reduction in hospital readmission as compared to the general 90-day readmission risk in SMI individuals during the risk period.

## Materials and methods

Data source

The Detroit Wayne Integrated Health Network (DWIHN) is a payor and service provider for one of the counties in Michigan. DWIHN is responsible for all behavioral health services provided to individuals with SMI and has claims data for all inpatient as well as outpatient services for the Medicaid population (including clubhouse claims). The source of DWIHN's data is based on approximately 250,000 individuals' demographic dataset in DWIHN's Data Warehouse. This dataset includes members from 2012 to the present (February 2025) and presently does not exclude inactive members or those who registered but did not receive services. The Information Technology (IT) Department assisted with the data collection by creating a report that had details of the inclusion and exclusion criteria of the study as described below without the use of a tool. There could be some claims that may have been rejected by the system due to submission errors, which is a data limitation. 

This was a retrospective study using de-identified aggregate claims data. 

Selection process

**Figure 1:**
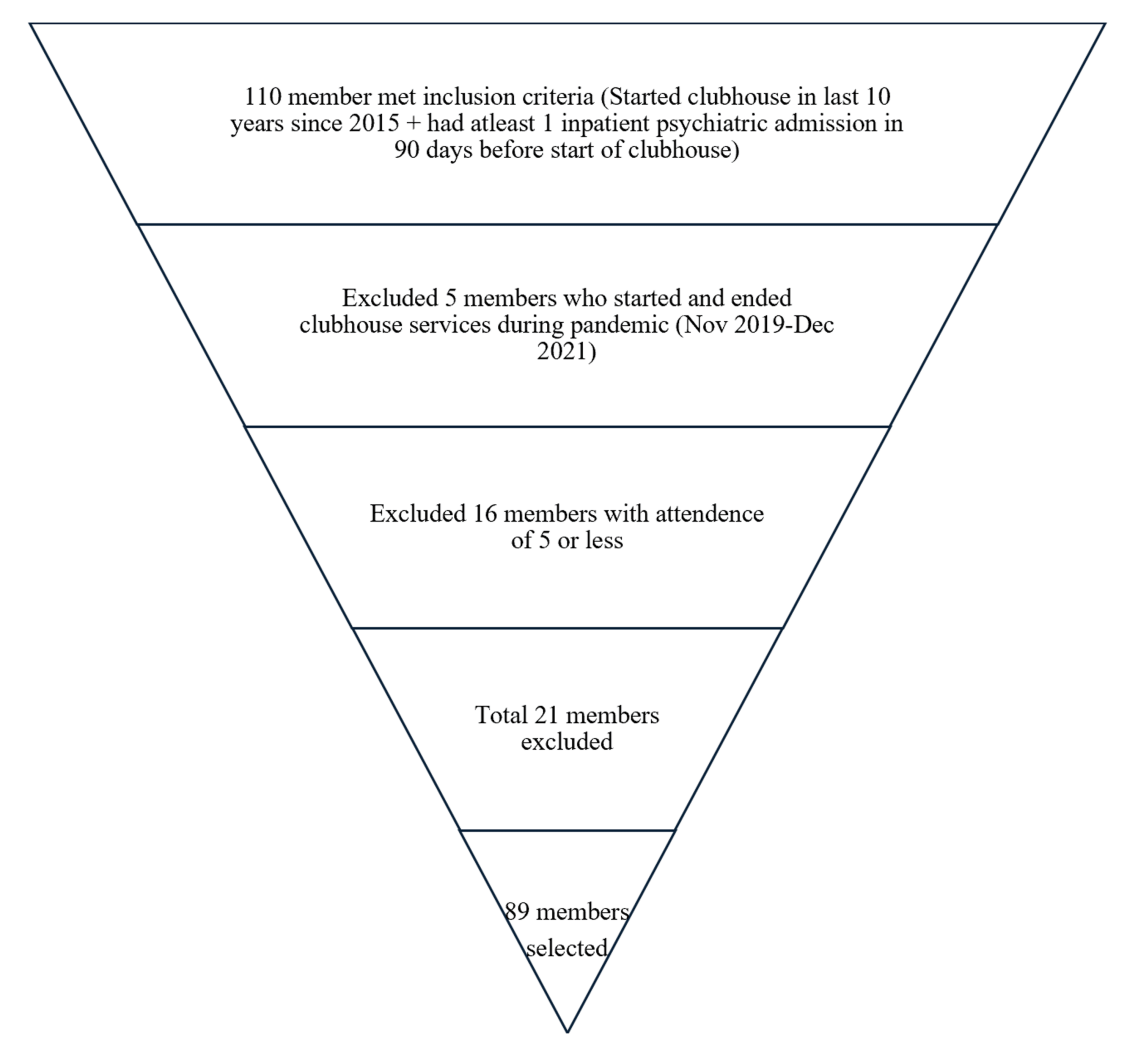
Selection process

Purposive sampling was used to strategically select cases to meet the study's inclusion criteria. For the inclusion criteria, those who attended clubhouse in the last 10 years (2015 or after) and had a psychiatric admission within 90 days before starting clubhouse were included. In contrast, for the exclusion criteria, those with an attendance of five or fewer days at clubhouse and who attended (started and ended) clubhouse only during the pandemic (i.e., enrolled between November 2019 and discharged before December 2021) were excluded from the study (Figure 1).

The Research Advisory Committee (RAC) of DWIHN reviewed the study and the article, and it was approved on 02/14/2025 with an approval number of 041123.

Data analysis

Eighty-nine members were selected using the selection criteria and process, and their hospitalizations were calculated 90 days before and after clubhouse enrollment. Significance was established at <0.05. A two-tailed t-test was performed for statistical analysis and to determine if the pre- and post-enrollment measurements were statistically significant and if the reduction in psychiatric admissions was related to clubhouse participation. Calculations were done using the Social Science Statistics website.

The 90-day readmission rate for clubhouse members was also calculated and compared to DWIHN's general 90-day readmission rate. 

## Results

**Figure 2:**
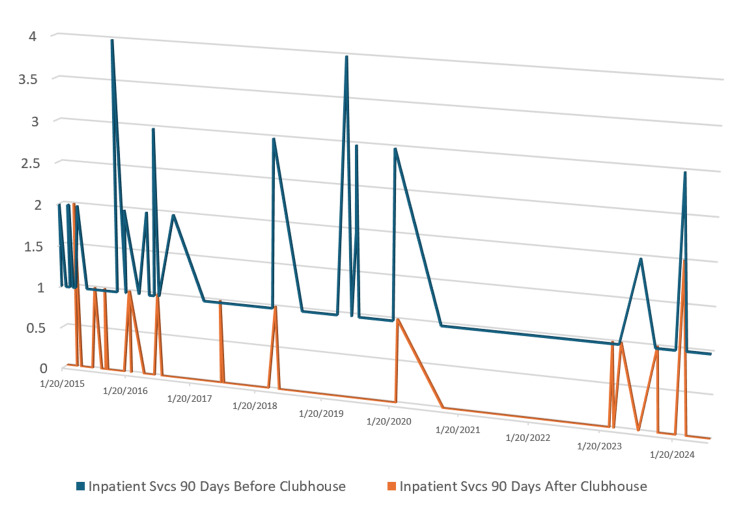
Change in inpatient psychiatric admissions after clubhouse engagement The data represents the number of hospitalization(s) (N) for each patient on the y-axis and the timeframe of the hospitalization on the x-axis

Eighty-nine members were selected using the selection criteria and process. These members had enrolled in clubhouse during their "risk period" and had one or more psychiatric admissions in the 90 days preceding clubhouse enrollment. Collectively, the 89 members had a total of 114 psychiatric admissions prior to starting clubhouse as some members had multiple admissions during that period. In the subsequent 90 days, 79 of those 89 members showed a reduction in the number of psychiatric admissions after starting clubhouse and attending it for more than five days during the "risk period," nine members had no change in hospitalizations, and one member had an increase in hospitalization (Figure 2).

Seventy-nine members had a reduction in hospitalizations from 104 before enrollment to four after enrollment. Nine members stayed with one hospitalization each before and after clubhouse enrollment, and one member had two hospitalizations after clubhouse enrollment versus one pre-enrollment. Collectively, the same 89 members who had 114 admissions before starting clubhouse had 15 psychiatric admissions in the 90 days post-clubhouse enrollment. A significant reduction in psychiatric readmission rates was observed, with an eightfold decrease in hospitalizations post-clubhouse enrollment (p<0.00001). A two-tailed t-test was performed to determine if the reduction in psychiatric admissions was related to clubhouse participation. The value of t is calculated at -13.801, and the value of p is less than 0.00001 (p<0.05) (Table 1). Therefore, the observed difference between the pre- and post-enrollment measurements is statistically significant, indicating a notable effect of clubhouse. 

**Table 1:** Hospitalization data before and after clubhouse p<0.00001 (extremely statistically significant)

Hospitalizations (total) (N= 89)	Hospitalizations (decreased) (N=79)	Hospitalization (no change) (N=9)	Hospitalizations (increased) (N=1)	Mean hospitalization	M (difference in mean)	df	Squared deviation	Two-tailed t-value	P-value
Before clubhouse	104	9	1	1.280898876					
After clubhouse	4	9	2	0.168539326					
Analysis					0.167-1.28=-1.11	88	50.88	-13.801	<0.00001 (the result is significant at p<0.05)

The 90-day readmission rate for clubhouse members was calculated to be 13.15% (114 baseline admissions and 15 readmissions). It was considered that not all members have a risk of readmission after a previous psychiatric admission; therefore, a general 90-day readmission rate was calculated for the "control group" for the last five years (Table 2). This was calculated using the 90-day readmission rate for all DWIHN members who were getting hospitalized each year. 

**Table 2:** 90-day psychiatric readmission rate for the general Medicaid population for five fiscal years

Fiscal years	90-day recidivism rate
2020	30.3%
2021	27.3%
2022	26.6%
2023	26.2%
2024	28%
5-year average	27.68%

**Figure 3:**
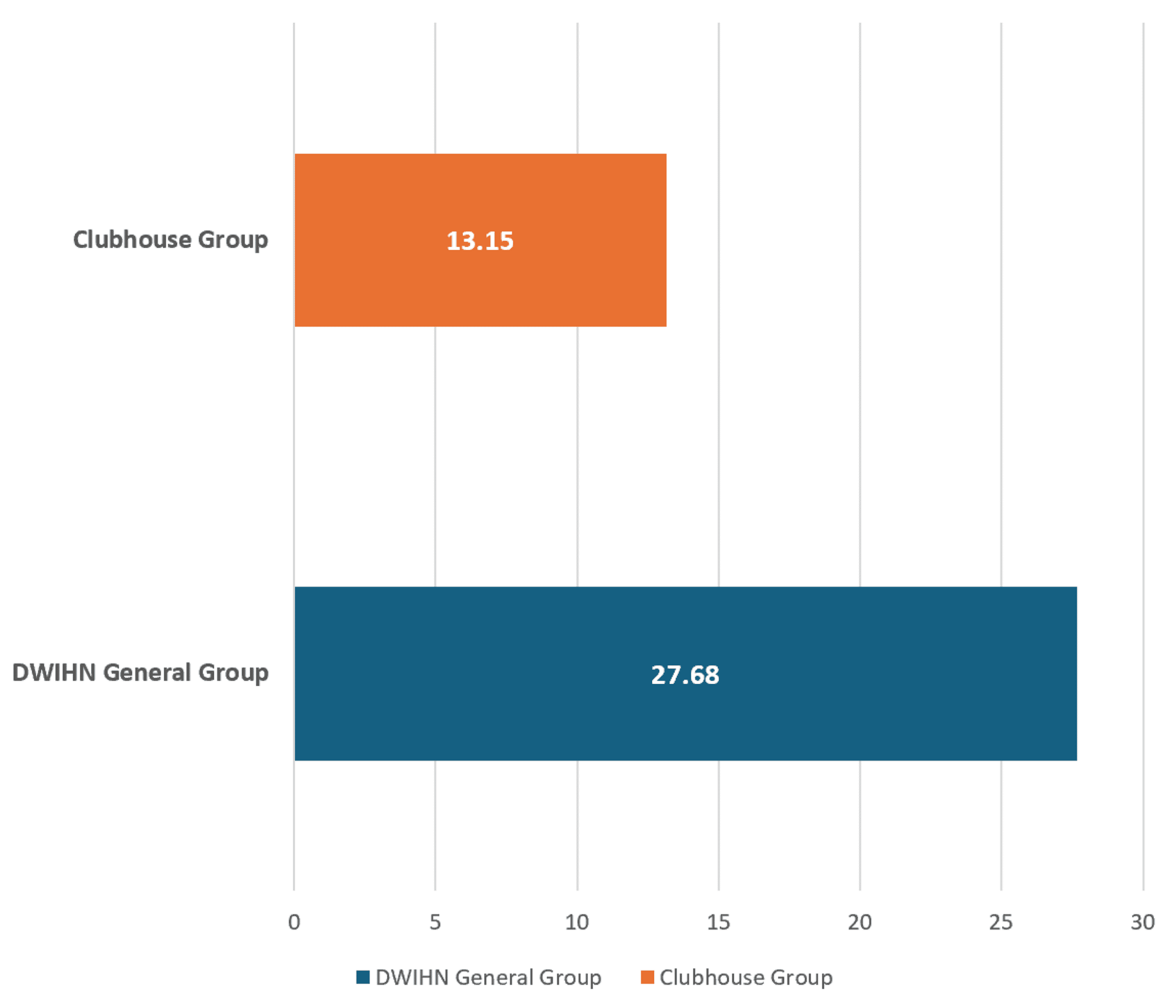
90-day recidivism rate for the clubhouse group versus the general inpatient psychiatric hospitalization group The data on the chart indicates the 90-day recidivism (readmission rate) as a percentage (%) marked on the x-axis DWIHN: Detroit Wayne Integrated Health Network

The average of the last five years was calculated to be 27.68%. Comparing the 90-day recidivism rate of members attending clubhouse to the 90-day recidivism rate of general DWIHN members, we found a more than 50% reduction in their risk of readmission to a psychiatric hospital (Figure 3).

## Discussion

The SMI population is at high risk of readmission within 30, 60, and 90 days of psychiatric admissions [2]; therefore, interventions that could address and decrease the recidivism rate are of great significance to service providers and payors.

Clubhouse models have existed for more than 65 years since the first one "Fountain House" started in New York [15]. A clubhouse consists of the voluntary participation of adults and young adults with mental illness and severe mental illness who contribute to its day-to-day work operations [16]. The persons living with mental illness are not treated as patients, and the staff ratio is intentionally kept low to have members work on accomplishing their jobs in a business-like team environment [5]. Clubhouse International oversees quality standards (International Clubhouse Standards) [17] that were developed to maintain uniformity and performance with over 370 clubhouses in 33 countries that are part of Clubhouse International [5].

A systematic review that looked at the quality outcome for accredited clubhouses reviewed at least 10 studies that looked at hospitalization as an outcome, and at least six of them found some support for a reduction or delay in the rehospitalization for the attendees with decreased recidivism rates and/or shorter hospital stays [10]. However, most of those studies have been more than a decade old, and recent data looking at the effect of clubhouses on psychiatric hospitalization and readmission is limited [11].

DWIHN, being the largest community mental health service provider in Michigan, serves 123,000 adults and children in Wayne County and typically spends a large amount of its annual budget on inpatient psychiatric hospitalization [18]. A decrease in the number of psychiatric admissions, typically measured over 30 days, 90 days, or one year, is an important quality goal for health plans and providers [19]. Using claims data for the last 10 years, we were able to identify individuals who enrolled in clubhouses within 90 days of one or more psychiatric admissions. We found a statistically significant decrease in the hospitalizations (eightfold) in the subsequent 90 days after starting and attending clubhouse. The readmission rate for members attending clubhouse was 13.15% as compared to a five-year average 90-day readmission rate of 27.68% for all DWIHN population indicating a 50% reduction in recidivism rate for members attending clubhouses. Though previous studies have also shown a reduction in psychiatric admissions and recidivism [10-14], many of those studies have either not been recent or have not seen such a significant reduction. The data submitted is evidence to support the conclusion that clubhouse services have been effective in reducing psychiatric admissions. This updated study shows a definitive benefit to these services being included in treatment plans for individuals in psychological recovery.

A study doing a comparative cost analysis of clubhouses found them to be highly cost-effective, with one year of recovery services costing the same as a two-week psychiatric hospital stay and much less than community mental health centers and assertive community treatment models [20]. Another one compared the cost of members of the same clubhouse who attended it more than three days a week and compared them to the ones who attended less often and found a significantly lower annual cost of care for members who had higher clubhouse attendance indicating potential healthcare savings with increased clubhouse participation [21].

Clubhouses provide psychological recovery to individuals with mental illness by providing them with employment and education, development and enhancement of their skills, and an overall sense of wellness and belonging [5-8]. Clubhouses can also support individuals in their continued recovery following an inpatient psychiatric stay and contribute to reducing the risk of readmission.

Strengths, implications, and future directions

The most important strength of our study is adding this to the limited literature that tracked clinical outcomes for clubhouse participants. Even with its retrospective design, the re-demonstration of some previous findings of decreasing recidivism is a great strength of this study, given the high psychiatric recidivism rates in the SMI population. 

This study holds future significance for us and similar organizations working with the SMI population, as it aims to promote the enrollment of high-risk individuals in clubhouses following psychiatric admissions and is especially critical for individuals who experience frequent rehospitalizations. We also aim to encourage strong attendance, particularly within the first 30-90 days, when members are most vulnerable to readmission. Once these objectives are met, we will assess the potential for expansion. While this study focused on collective data from all clubhouses, we plan to analyze individual clubhouse data to determine if any one location demonstrates better outcomes, with an emphasis on the quality of services provided.

Limitations

The limitation of our study is its retrospective design. Despite doing data and statistical analysis, there could have been other factors such as medication adherence, family support, outpatient therapy engagement, and other factors that may have also contributed to and impacted the data, recovery, and outcomes as it was not a controlled study.

Though we excluded the members who started and ended services only during the pandemic years, there were still members who continued services during the pandemic years, and clubhouse services were either closed, partially closed, or virtual for some part of the pandemic which may have impacted some outcomes and data.

There could be some claims that may have been rejected by the system due to submission errors which is a data limitation. 

## Conclusions

Clubhouses have an effective role not only in rehabilitation but also in reducing psychiatric hospitalization and readmission during the high-risk period. Our research indicated a reduction in psychiatric hospitalization and recidivism for clubhouse members in the 90 days after enrollment. For future studies, we plan to extend the data to observe the continued benefits of clubhouse for the members and its continued impact on reducing psychiatric hospitalization past 90 days to look at long-term outcomes at six months and one year. We suggest additional studies that further look at the effect of clubhouse participation on hospitalizations in a controlled setting and explore its effects on reducing healthcare costs.
